# Fragment library screening identifies hits that bind to the non-catalytic surface of *Pseudomonas aeruginosa* DsbA1

**DOI:** 10.1371/journal.pone.0173436

**Published:** 2017-03-27

**Authors:** Biswaranjan Mohanty, Kieran Rimmer, Róisín M. McMahon, Stephen J. Headey, Mansha Vazirani, Stephen R. Shouldice, Mathieu Coinçon, Stephanie Tay, Craig J. Morton, Jamie S. Simpson, Jennifer L. Martin, Martin J. Scanlon

**Affiliations:** 1 Medicinal Chemistry, Monash Institute of Pharmaceutical Sciences, Monash University, Parkville, Victoria, Australia; 2 The University of Queensland, Institute for Molecular Bioscience, Division of Chemistry and Structural Biology, Brisbane, Queensland, Australia; 3 Biota Holdings Limited, Notting Hill, Victoria, Australia; Francis Crick Institute, UNITED KINGDOM

## Abstract

At a time when the antibiotic drug discovery pipeline has stalled, antibiotic resistance is accelerating with catastrophic implications for our ability to treat bacterial infections. Globally we face the prospect of a future when common infections can once again kill. Anti-virulence approaches that target the capacity of the bacterium to cause disease rather than the growth or survival of the bacterium itself offer a tantalizing prospect of novel antimicrobials. They may also reduce the propensity to induce resistance by removing the strong selection pressure imparted by bactericidal or bacteriostatic agents. In the human pathogen *Pseudomonas aeruginosa*, disulfide bond protein A (PaDsbA1) plays a central role in the oxidative folding of virulence factors and is therefore an attractive target for the development of new anti-virulence antimicrobials. Using a fragment-based approach we have identified small molecules that bind to PaDsbA1. The fragment hits show selective binding to PaDsbA1 over the DsbA protein from *Escherichia coli*, suggesting that developing species-specific narrow-spectrum inhibitors of DsbA enzymes may be feasible. Structures of a co-complex of PaDsbA1 with the highest affinity fragment identified in the screen reveal that the fragment binds on the non-catalytic surface of the protein at a domain interface. This biophysical and structural data represent a starting point in the development of higher affinity compounds, which will be assessed for their potential as selective PaDsbA1 inhibitors.

## Introduction

*Pseudomonas aeruginosa*, an opportunistic and naturally multi-drug resistant Gram-negative bacterial pathogen, is a frequent causative agent of acute nosocomial infection and chronic infection in individuals with cystic fibrosis. An ESKAPE pathogen [1] it is feared that *P*. *aeruginosa* may eventually become resistant to all currently available antibiotics. Accordingly new therapeutic agents and strategies to counter infection are urgently sought.

Enzymes that catalyze disulfide bond formation in the periplasm of many Gram-negative bacteria are essential for virulence [2]. This family of disulfide oxidoreductases, in particular the primary oxidase DsbA and its partner membrane-protein DsbB, co-operate to catalyze the oxidative folding of disulfide bond containing proteins, many of which are secreted or cell-surface virulence factors. Deletion of DsbA in pathogenic Gram-negative bacteria prevents lethal infection in many animal models [3, 4] and has pleiotropic effects on virulence [2]. Deletion of *P*. *aeruginosa dsbA1* (that encodes PaDsbA1) elicits numerous effects in phenotypic assays including: inability to process elastase to a mature form [5]; inability to export the Type 3 Secretion System (T3SS), and toxins ExoU [6] or ExoT [6, 7]; impaired ability to survive intracellularly in HeLa cells [6]; loss of twitching motility [6, 8]; and reduced alkaline phosphatase and lipase activity [8]. Consequently PaDsbA1 is a highly significant control point affecting the function of many downstream virulence effector molecules and as such is a potential target for inhibitor development.

We screened an in-house fragment library [9] and identified small molecules that bind to PaDsbA1. Analysis of their binding mode using protein-detected NMR spectroscopy suggested that several of these fragments bind PaDsbA1 on the face opposite the active site surface. This is in contrast to prior work with *Escherichia coli* DsbA (EcDsbA) where structures of co-complexes revealed fragments that bound in a groove adjacent to the active site [10]. Consistent with the different binding location of the fragments to the respective proteins, there was very little overlap between the set of fragments that were identified as binding to PaDsbA1 and fragments that bound to EcDsbA. To characterize the structure of a PaDsbA1—fragment complex in more detail we utilized both NMR spectroscopy and X-ray crystallography. The structures of the co-complex that were generated in the two approaches provided complementary information and confirmed that the fragment bound on the non-catalytic surface of the protein.

## Materials and methods

### Protein expression and purification

The *P*. *aeruginosa dsbA1* gene lacking its signal sequence was inserted into a modified pET28a plasmid (a derivative of pET28a (Novagen)) to generate a construct with a Tobacco Etch Virus (TEV) protease cleavable N-terminal His_6_-tag as described [11].

To generate protein for the NMR experiments, PaDsbA1 was expressed and purified essentially as described [11]. Transformed BL21(DE3)-Gold *E*. *coli* were grown at 37°C in Luria Broth to produce unlabeled protein, or at 28°C in isotopically enriched M9 minimal media to produce isotopically labeled protein [10]. Purification involved successive steps of immobilized metal ion affinity chromatography (IMAC, HisTrap column, GE Healthcare) followed by hydrophobic interaction chromatography (PhenylHP column, GE Healthcare) prior to His_6_-tag removal by TEV protease treatment [12]. A second IMAC step removed uncleaved PaDsbA1 and TEV protease. PaDsbA1 was oxidized with a either a 50:1 molar excess of oxidized glutathione or by addition of freshly prepared copper phenanthroline to a final concentration of 1.5 mM and purified by size exclusion chromatography as described [10, 13].

For crystallization experiments a PaDsbA1 variant was employed. This variant was engineered to overcome crystal-packing interactions between His39 of the active site and Glu82_sym_ of a symmetry related molecule, which impeded access to the active site of PaDsbA1. In the variant Glu82 was mutated to Ile to enable fragments to access the active site upon soaking into crystals of PaDsbA1 [13]. PaDsbA1 Glu82Ile was expressed and purified as described [13]. EcDsbA was expressed and purified as described previously [10].

### Fragment library screening

Fragment binding was assessed by recording saturation transfer difference (STD) NMR experiments [14] in cocktails containing up to 6 individual fragments. The mixtures contained PaDsbA1 at a concentration of 5 μM in 50 mM HEPES, 50 mM NaCl, pH 7.4, 10% ^2^H_2_O, 1% ^2^H_6_-DMSO. Each of the samples contained a unique combination of up to 6 library fragments, with each fragment at a concentration of ~330 μM. The fragments were combined to minimize overlap in their 1D ^1^H-NMR spectra and to allow facile identification of binders within each cocktail [9]. STD NMR experiments [14] were conducted at 10°C and 600 MHz on a Bruker Avance spectrometer equipped with CryoProbe. The magnitude of the signal in STD spectra was ranked by comparison with the most intense STD signal (I_max_) identified across all the STD spectra for PaDsbA1 as previously described [15]. The STD signal was categorized as strong where the intensity was ≥75% I_max_, medium where the intensity was ≥50% I_max_ and <75% I_max_ and weak where the intensity was ≥25% I_max_ and <50% I_max_. All fragments for which weak or better signals were observed in the STD-NMR spectra were retained for further analysis.

### Validation of binding to PaDsbA1

Fragments for which binding was observed in the STD NMR screen of the library mixtures were validated by recording ^15^N-heteronuclear single quantum coherence (HSQC) spectra of uniformly ^15^N labeled PaDsbA1 (100 μM) in 50 mM phosphate buffer, containing 50 mM NaCl, pH 7.4, 10% ^2^H_2_O, 1.7% (*v/v*) ^2^H_6_-DMSO in the absence and presence of each individual fragment (3.3 mM). ^15^N-HSQC experiments were recorded at 25°C and either 600 or 800 MHz on Bruker Avance spectrometers equipped with a CryoProbe. Weighted chemical shift perturbations (CSP) that were observed upon addition of fragments to PaDsbA1 were calculated using Eq 1 [16]:

$$CSP=\;\sqrt{\Delta \delta_{H}{}^{2}+{\left(0.2\times \Delta \delta_{N}\right)}^{2}}$$ (1)

Where Δδ_H_ and Δδ_N_ denote the changes in chemical shift of proton and nitrogen resonances upon addition of the fragment. Peak assignment for measurement of CSPs was performed with the program Sparky (Goddard & Kneller, SPARKY 3, University of California, San Francisco). Equilibrium dissociation constants (K_D_) were estimated by fitting the magnitude of CSP observed in ^15^N-HSQC spectra upon titration of PaDsbA1 with increasing concentrations of fragment to a one-site binding model (Eq 2), which accounts for ligand depletion using GraphPad Prism 6.0 (GraphPad, La Jolla, CA)

$$CSP=\;\frac{CSP_{max}\times \;{\left[\left(K_{D}+\;P_{t}\;+\;L_{t}\right)\;-\;\sqrt{\left({\left(K_{D}+\;P_{t}+\;L_{t}\right)}^{2}\;-\;4\;\times \;P_{t}\;\times \;L_{t}\right)}\right]}_{}}{2\times P_{t}}$$ (2)

Where CSP is the measured CSP at a given fragment concentration; CSP_max_ is the CSP observed upon saturation of the protein with fragment, P_t_ and L_t_ are the total protein and ligand concentrations, respectively at each point in the titration. The experimental K_D_ values for fragment binding were used to generate ligand efficiency and lipophilic ligand efficiency metrics as described previously [17]. The ligand efficiencies are expressed in units of kcal mol^-1^ divided by the heavy atom count (HAC) for the fragment.

### Fragment binding to EcDsbA

Fragment binding to EcDsbA was measured by recording ^15^N-HSQC spectra for EcDsbA in the absence and presence of fragments. Experiments were recorded at 25°C in a buffer system of 50 mM HEPES, 50 mM NaCl, pH 6.8, 10% ^2^H_2_O, 0.5% ^2^H_6_-DMSO.

### Chemical shift assignment and NMR solution structure of oxidized PaDsbA1

A sample of uniformly ^13^C,^15^N labelled oxidized PaDsbA1 (2 mM) was prepared in 50 mM phosphate, 50 mM NaCl, pH 7.4, 10% ^2^H_2_O. NMR experiments were conducted at 25°C and 600 or 800 MHz on Bruker Avance spectrometers equipped with a CryoProbe. H^N^, N, C^α^, C^α-1^, C^β^, and C^β-1^ peak lists were generated manually with the program CARA [18] using 2D ^15^N-HSQC, 3D HNCA, 3D CBCA(CO)NH and 3D HNCACB spectra and used as the input for automated backbone assignments in UNIO-MATCH [19]. The automated assignments were checked manually and extended using a 3D ^15^N-resolved NOESY. H^β^, H^α^ assignments were obtained using a 3D HBHA(CBCACO)NH spectrum. H^N^, N, C^α^ and C^β^ assignments together with H^β^, H^α^ were provided as input for UNIO-ATNOS/ASCAN for automated side-chain assignments using 3D ^15^N-, ^13^Cali—and ^13^C_aro_—resolved NOESY datasets [20]. Finally, all assignments were evaluated and extended by manual inspection. In addition, a selectively ^13^C-enriched sample of PaDsbA1 was prepared and used for stereospecific assignment of prochiral methyl groups of valine and leucine sidechains [21]. Upper distance constraints for structure calculations were automatically generated from the NOESY data using UNIO-ATNOS/CANDID [22, 23] and the structure was solved using the torsion angle dynamics program CYANA3.0 [23]. The conformers having the lowest CYANA target function values were energy minimized with OPALp [24] and their quality assessed using structure validation tools (http://www.pdb.org/ and http://www.nihserver.mbi.ucla.edu/). Structures were inspected and analysed with MOLMOL [25]. The coordinates of the solution NMR structure of *apo*-oxidised PaDsbA1 were deposited in the Protein Data Bank with accession number 2MBT.

Backbone assignments for PaDsbA1 were obtained using a semi-automated approach. In this way, 163 of 178 expected backbone amide resonances and 87% of all possible PaDsbA1 proton resonances were successfully assigned. A number of backbone amides and side chain resonances for PaDsbA1 were not assigned in the spectra including residues Glu87, His88, Asp89, Val90, His91 and Phe95 within the helical domain. Attempts to improve the number of observable resonances by altering experimental conditions (temperature and pH) were not successful. Nonetheless, the extent of assignments gave sufficient coverage of the protein structure to probe the binding location of the fragments.

### Chemical shift assignment for Fragment 1

The proton chemical shifts of Fragment **1** in its free state were assigned using standard 1D and 2D experiments including: 1D ^1^H, 2D ^13^C-edited HSQC, HMBC, H2BC and 2D [^1^H,^1^H]-NOESY spectra. The proton chemical shift assignments of Fragment **1** in the bound state were obtained by recording 1D ^1^H-NMR spectrum of the complex in the presence of increasing concentrations of Fragment **1** and tracking the chemical shifts from their assigned resonance positions in the free state.

### Solubility of Fragment 1 in aqueous NMR buffer

A 2D [^1^H,^1^H]-NOESY was recorded for Fragment **1** in aqueous NMR buffer (50 mM phosphate, 50 mM NaCl, pH 7.4) at 3.3 mM in the absence of protein. In this spectrum the cross peaks were found to have the opposite sign to the diagonal, indicating that the fragment was soluble and did not aggregate under the experimental conditions.

### Solution structure of the complex of PaDsbA1 bound to Fragment 1

To generate a model of the solution structure of the PaDsbA1–ligand complex a sample of uniformly ^13^C, ^15^N labelled oxidized PaDsbA1 (300 μM) was prepared in a buffer of 50 mM phosphate, 50 mM NaCl, pH 7.4, 100% ^2^H_2_O. Fragment **1** was added to a final concentration of 3.3 mM and the resulting sample contained 1.7% (*v/v*) ^2^H_6_-DMSO. NMR experiments were conducted at 25°C and 600 MHz or 800 MHz on Bruker Avance spectrometers equipped with a CryoProbe. Assignments for side chain methyl resonances in the complex were obtained by recording a series of ^13^C-HSQC spectra in the presence of increasing concentrations of Fragment **1** to track the CSPs. Distance constraints were derived from intermolecular NOEs observed in 3D ω1-^13^C,^15^N-filtered, ω3-^13^C_ali_ edited [^1^H,^1^H]-NOESY datasets acquired on the complex.

Structures of the PaDsbA1–Fragment **1** complex were calculated using HADDOCK [26, 27]. The twenty conformers representing the NMR solution structure of *apo* PaDsbA1 were used as starting structures in HADDOCK calculations. Conformations of **1** were generated using Maestro (Schrödinger 2014) and the two lowest energy conformers were selected as input for the docking calculations. Topology and parameter files for the Fragment **1** were generated using an automated topology force field builder (ATB) server [28, 29]. Intermolecular NOEs observed in the experimental data were converted to distance restraints for the structure calculation as described previously [30]. The NOE distances were used as unambiguous restraints in the HADDOCK calculations. Due to lower resolution in the indirect 3D ω1-^13^C,^15^N-filtered, ω3-^13^C_ali_ edited [^1^H,^1^H]-NOESY dimension, intermolecular NOE cross peaks originating from ligand H4, H5 and H6 proton signals could not be resolved in the data. Therefore a single target distance constraint was set between protein methyl and H4/H5/H6 protons. In addition to these NOE-derived distance restraints, surface exposed residues of PaDsbA1 that displayed significant CSPs in ^13^C-HSQC spectra upon addition of Fragment **1** were designated as ambiguous interaction restraints in the HADDOCK calculation. Standard rigid body HADDOCK docking was used to generate 1000 models of the complex. The 200 models with the best HADDOCK scores were taken for semi-rigid body simulated annealing in torsion angle space followed by water refinement in Cartesian space. Finally, the top 10 models of the complex with the best HADDOCK scores were retained for analysis and deposited in the Protein Data Bank with accession number 2MBU.

### Crystallisation, X-ray data collection and structure determination and refinement

Our initial efforts to determine structures of fragment complexes using X-ray crystallography were complicated by our observation that in the wild-type PaDsbA1 crystal structure there is an unfavourable crystal-packing interaction, which appears to preclude access to the active site of the protein. Consequently, for the purpose of all crystal soaking experiments, we used a protein variant PaDsbA1 E82I, which contains a single amino acid substitution (Glu 82 to Ile) introduced to disrupt this interaction [13]. Importantly PaDsbA1 E82I displays unaltered *in vitro* activity, redox character and only minor structural differences relative to wild-type PaDsbA1. PaDsbA1 E82I was crystallized as described previously [13]. For fragment soaks, PaDsbA1 E82I crystals were immersed for several minutes in solutions of single fragments at a final concentration of 20 mM or 50 mM, and a maximum DMSO concentration of 10% (v/v), immediately prior to cryoprotection.

Crystals were cryocooled in liquid nitrogen using mother liquor and 20% glycerol as a cryoprotectant. X-ray data were collected at 100 K at the Australian Synchrotron on the microfocus beamline MX2 using an ADSC Quantum 315r detector. Data were collected at a wavelength of 0.95370 Å over a total angular rotation of 180° using 1° phi slices and an exposure time of 1 s. The data were indexed and integrated with iMosflm [31], and scaled using POINTLESS and AIMLESS [32] within the CCP4 suite.

All phasing and model refinement procedures were implemented within the Phenix software suite. The data were phased using molecular replacement methods with AutoMR [33] using PaDsbA1 (PDB ID, 4ZL7) as a search model. The resulting model was subject to iterative rounds of refinement (phenix.refine [34])—including initially refinement using TLS groups and of hydrogens using a riding model—and model building using COOT [35]. In the final stages of refinement non-water atoms were refined with anisotropic atomic displacement parameters. Coordinates and restraints for Fragment **1** as well as bound ligands derived from the cryoprotectant (glycerol), and crystallisation condition (MES) were generated using elBOW [36] and the quality of the final model was assessed with Molprobity [37] throughout the refinement process. The R-factor was calculated according to Eq 3:

$$Rfactor=\sum_{h}∥{F_{obs}|}_{h}-{|F_{calc}|}_{h}|/\sum_{h}|{F_{obs}|}_{h}$$ (3)

Where h defines the unique reflectons.

In the final model there are continuous regions of density near residues 69–72 (helix 2) and 180-181(helix 7). Efforts to model and refine sensible ligands (e.g. components of the crystallisation condition or soak) were unsuccessful. These regions were modelled as waters. The final refined structure has been deposited in the Protein Data Bank (PDB ID 5DCH). All structural figures were generated using Pymol (PyMOL Molecular Graphics System, version 1.6 Schrodinger, LLC, http://pymol.org) and Adobe Illustrator.

## Results

### Fragment library screening

**Fig 1:**
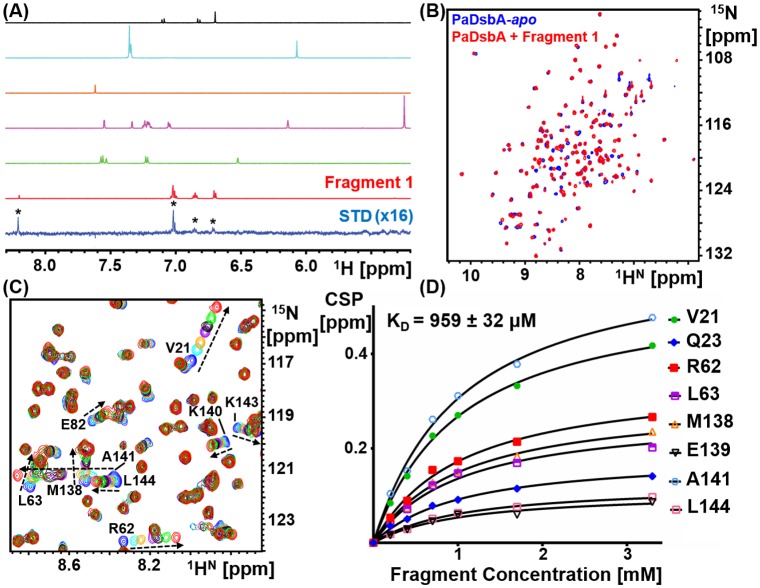
Screening and validation of the fragment library. **(A)** The fragment library was screened using ligand-detected ^1^H STD NMR. Pictured is an overlay of the aromatic ^1^H region of an STD spectrum (bottom spectrum, blue), plus the 1D reference spectra of fragments present in the cocktail (red, green, magenta, orange, cyan and black). In this instance, of the fragments present in the cocktail, only Fragment **1** whose spectrum is shown in red binds to PaDsbA1. STD fragment hits were validated by recording HSQC NMR using ^15^N labeled PaDsbA1. (**B**) Pictured is an overlay of the ^15^N-HSQC spectra of PaDsbA1 in the absence (blue) and presence (red) of Fragment **1**, which reveals perturbation of a subset of residues. (**C**) The K_D_ for each fragment was estimated from measurement of chemical shift perturbations in ^15^N-HSQC spectra of PaDsbA1 as a function of increasing fragment concentration. Shown is an overlay of one region of the ^15^N-HSQC spectra of PaDsbA1 in the absence (blue) and then increasing concentrations of Fragment **1** (0.2–3.3 mM). (**D**) The concentrations dependent chemical shift perturbation data were fitted to a one-site binding model to estimate a K_D_ for the compound.

PaDsbA1 was screened against a library of 1137 fragments [9] by ligand-detected STD NMR experiments (Fig 1A). A total of 215 fragments, (~19% of the library) were classified as hits in this screen, whereby at least one well resolved resonance in the NMR spectrum of the fragment gave an STD above the detection threshold. These fragments were subsequently validated using protein-detected NMR analysis of binding.

### Hit validation and assessment of specificity for PaDsbA1

**Fig 2:**
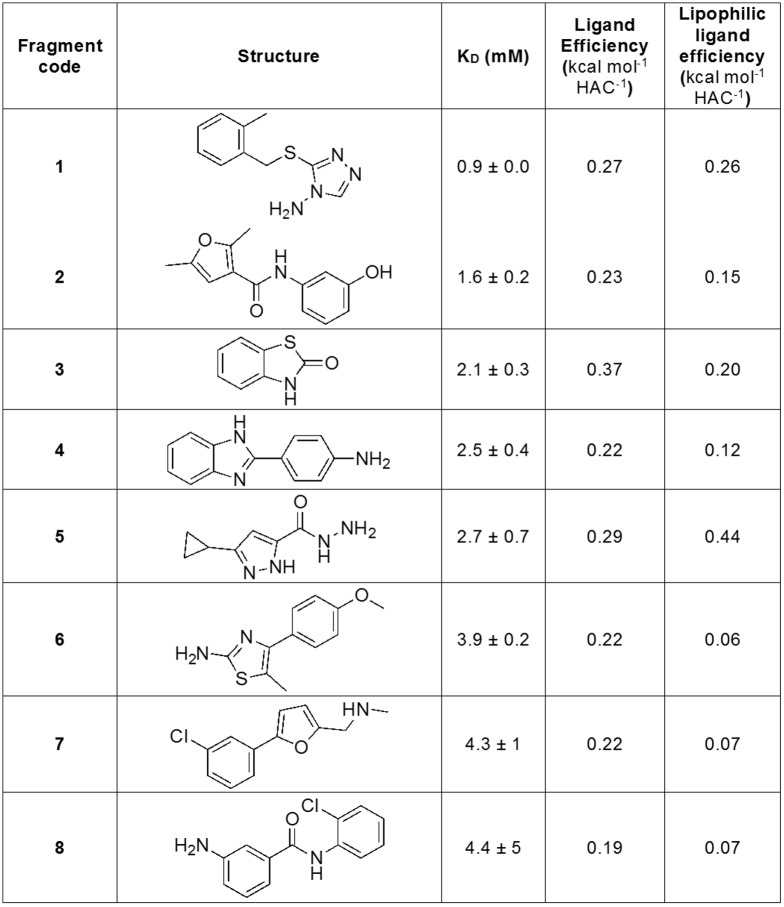
Validated fragment hits with K_D_ values < 5 mM.

Of the STD hits, a total of 45 fragments caused a weighted chemical shift perturbation (CSP) of at least 0.01 ppm for one or more peaks in the ^15^N-HSQC spectrum of PaDsbA1 at a fragment concentration of 3.3 mM (an example of which is illustrated in Fig 1B). This represents 21% of the STD hits and 4.0% of the overall fragment library. ^15^N-enriched PaDsbA1 was titrated with each of these 45 fragments to generate a concentration-dependent series of CSP in ^15^N-HSQC spectra and, where possible, the CSP data were used to estimate the K_D_ for the fragment (Fig 2). Representative HSQC spectra and curve fitting for titration experiments are shown for Fragment **1** in Fig 1C and 1D. K_D_ values were obtained for 16 fragments by this method and 8 of these gave fitted K_D_ values of less than 5 mM (Fig 2).

To assess the binding selectivity of the 8 fragments that bound to PaDsbA1 with the highest affinity, we used the same binding screen and validation protocol outlined above (ligand-detected STD NMR followed by protein-detected ^15^N-HSQC NMR), to assess their binding to EcDsbA. These data indicated that of the 8 PaDsbA1-binding fragments, only Fragment **6** also binds to EcDsbA, and that the binding of **6** to EcDsbA was too weak (> ~5 mM) to allow estimation of its K_D_ by NMR. We have previously observed that 2-aminothiazoles such as Fragment **6** can be promiscuous binders in our NMR-detected fragment screens [38]. These findings suggest that the fragments that bind to PaDsbA1 with the highest affinity exhibit some selectivity for PaDsbA1 over EcDsbA.

### Identification of the fragment-binding site of PaDsbA1

**Fig 3:**
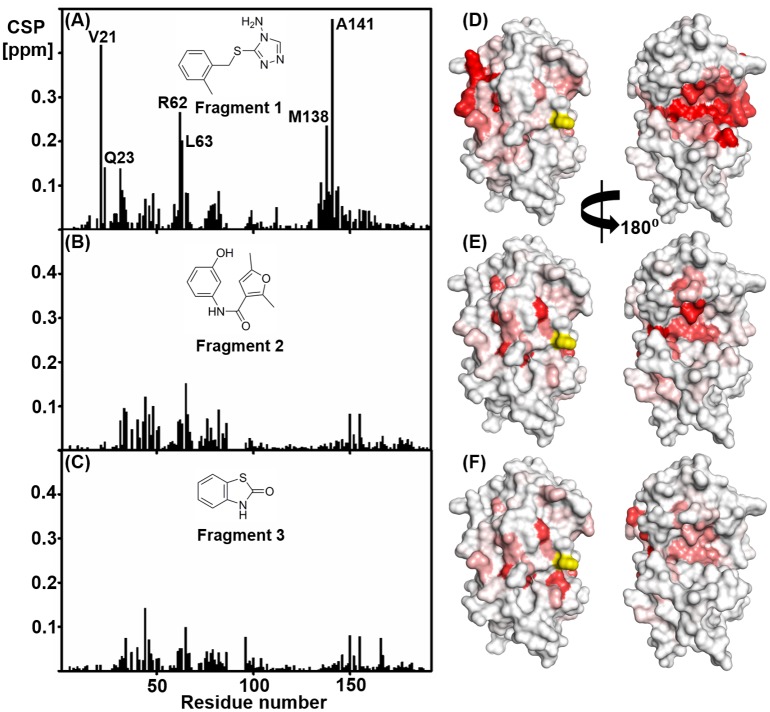
Chemical shift perturbations are observed for residues on both the catalytic and non-catalytic face of oxidised PaDsbA1. Chemical shift perturbations observed in HSQC spectra of PaDsbA1 in the presence of 3.3 mM (A) Fragment **1**, **(B)** Fragment **2** and **(C)** Fragment **3**. CSP are plotted versus residue number. (**D-F**) In each case the chemical shift perturbations have been mapped onto the crystal structure of PaDsbA1 (PDB code: 3H93): minimum = 0.01 ppm (white) and maximum = 0.1 ppm (red). Shown are views of the catalytic face of PaDsbA1 (left) and the non-catalytic face (right). In each case there is a continuous cluster of chemical shift perturbations observed on the non-catalytic face. The unassigned active site histidine residue (His39) is highlighted in yellow.

As with other DsbA enzymes, the catalytic surface of PaDsbA1 is composed of a CPHC active site, and three neighbouring surface loops that determine redox character as well as the nature of interactions with substrates and the partner protein DsbB [39, 40]. Titration with the most tightly binding fragments (**1–3**) resulted in the largest CSPs in HSQC spectra being observed for a cluster of residues that were not located on this active site surface but at the domain interface on the non-catalytic face of the protein, suggesting that this was the binding site for the fragments on PaDsbA1 (Fig 3). This is in contrast to our previous study with EcDsbA [10], where the fragments that we identified were found to bind in a hydrophobic pocket adjacent to the catalytic site.

### Structural characterization of the PaDsbA1–Fragment 1 complex

To investigate the location of fragment binding to PaDsbA1, we determined the structure of PaDsbA1 in complex with Fragment **1**. Our initial efforts to soak or co-crystallize PaDsbA1 with fragments proved unsuccessful, which led us to investigate the structure of the complex using NMR spectroscopy. Subsequently, using a single-point mutation of the protein [13] it was possible to generate crystals of the PaDsbA1–Fragment 1 complex and consequently, models of the co-complex structure were obtained using both NMR spectroscopy and X-ray crystallography.

For the NMR structure of the complex, we first determined the solution structure of oxidized PaDsbA1 using standard triple resonance NMR methods [41]. The 20 NMR conformers with the lowest energy and least residual violations of the experimental data were chosen to represent the solution structure of *apo*-PaDsbA1. This ensemble is a high precision structure with RMSD values for backbone and heavy atoms in the well-defined regions of the structure (Asp4–Gly85, Val94–His99 and Leu105–Lys192) of 0.74 ± 0.14 Å and 1.12 ± 0.14 Å, respectively. Statistics for the PaDsbA1 NMR structures are summarized in Table 1. Residues which fall into disallowed Ramachandran regions include the unassigned residues Asp4, His39, Ile70, His88, Val90 and His91 and residues Lys104 and Thr151, which are present in loop regions. The solution structure of PaDsbA1 is highly similar to that of the crystal structure of wild-type PaDsbA1 (PDB ID 3H93); the NMR conformers have a backbone RMSD of 1.72 ± 0.18 Å relative to the crystal structure of wild-type PaDsbA1 for the region Asp4–Gly85, Val94–His99 and Leu105–Lys192. The ensemble of 20 NMR conformers was used as a starting point for the structure determination of the complex.

**Table 1:** Structural statistics for the bundle of 20 energy-minimized NMR conformers of apo oxidised PaDsbA1 (3–192).

Quantity	Value^a^
Completeness of proton assignment	86.77%
NOE upper distance limits	3296
intraresidual	714
short-range	894
medium-range	777
long-range	911
Residual target function value [Å2]	2.4 ± 0.1
Residual NOE violations	
number ≥ 0.1 Å	25.9 ± 4.6
maximum [Å]	0.02 ± 0.0
Residual dihedral angle violations	
number ≥ 2.5°	0.4 ± 0.7
maximum [°]	2.2 ± 0.9
AMBER energies [kcal/mol]	
total	-6826 ± 111
van der Waals	-679 ± 19
electrostatic	-7568 ± 101
RMSD from mean coordinates^b^ [Å]	
backbone (4–85, 94–99, 105–192)	0.74 ± 0.14
all heavy atoms (4–85, 94–99, 105–192)	1.12 ± 0.14
Ramachandran plot statistics^c^	
most favoured regions [%]	82.2
additional allowed regions [%]	16.7
generously allowed regions [%]	0.6
disallowed regions [%]	0.5

^a^Except for the top entries (those relating to NOEs), average values and standard deviations for the 20 energy-minimized conformers are given. The top six entries represent the output generated in the final cycle of the UNIO-CYANA3.0 calculation.

^b^The numbers in parentheses indicate the residues for which the RMSD was calculated.

^c^As determined by PROCHECK.

**Fig 4:**
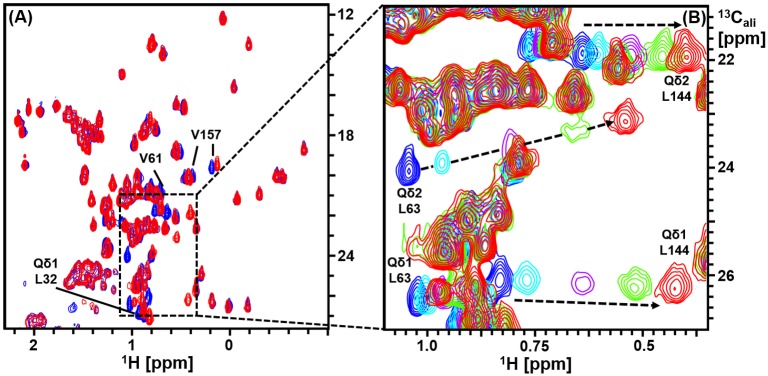
Assignment of methyl resonances of oxidized PaDsbA1 in the presence of Fragment 1. **(A)** Overlay of the methyl region of the ^13^C-HSQC spectra of PaDsbA1 in the absence (blue) and presence (red) of Fragment **1**. A subset of residues undergoes significant chemical shift perturbations. **(B)** Overlay of the ^13^C-HSQC spectra of PaDsbA1 in the absence (blue) and presence increasing concentrations of Fragment **1** (0.2–3.3 mM). Selected methyl resonance assignments are shown. The resonances for Leu63 and Leu144 undergo the largest chemical shift perturbations as indicated by arrows.

**Fig 5:**
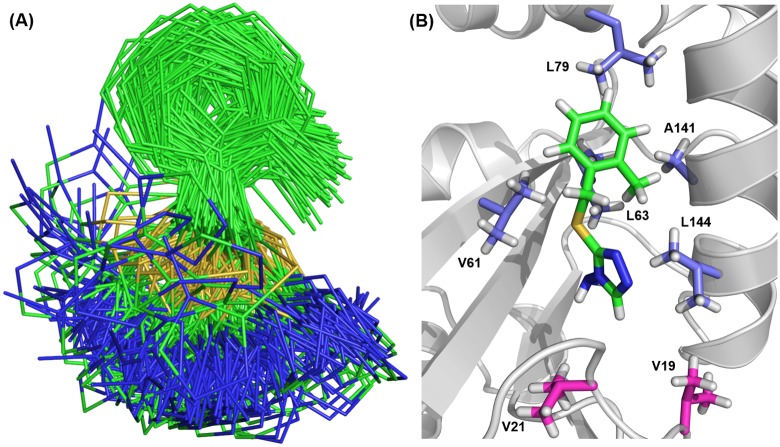
NMR data driven-HADDOCK docking model of PaDsbA—Fragment 1 complex. **(A)** Overlay of the two hundred best scoring HADDOCK model structures after water refinement showing the conformation of Fragment **1** in the complex. **(B)** The lowest energy conformer from the HADDOCK calculation is shown as a representative model of the PaDsbA—Fragment **1** complex. PaDsbA1 is shown in cartoon. Methyl containing residues for which intermolecular NOEs were detected, are shown in blue-white sticks. Val19 and Val21 methyls are present in close proximity of the Fragment **1** binding site (shown in magenta sticks), however no NOE cross peaks were observed from these methyls to ligand protons.

Intermolecular distance constraints derived from a 3D ω1-^13^C,^15^N-filtered, ω3-^13^C_ali_ edited [^1^H,^1^H]-NOESY spectrum were used to generate a model of the solution structure of the PaDsbA1—Fragment **1** complex. NOEs were observed involving all non-exchangeable protons of Fragment **1** with the exception of the single proton on the triazole ring, which was broadened beyond detection under the experimental conditions. A total of 23 NOEs were observed between methyl resonances of protein residues Val61, Leu63, Leu79, Ala141 and Leu144 and ligand resonances of Fragment **1**. CSPs of > 0.05 ppm in [^13^C, ^1^H]-HSQC were observed for most of the residues showing intermolecular NOEs (Leu79, Val61, Leu63 and Leu144), as well as for the adjacent buried residues Leu32 and Val157, plus the partially solvent exposed residue Val30 (Fig 4). These data were used to generate distance restraints for the HADDOCK calculation. In all, 200 HADDOCK models of the protein:ligand complex were generated, and in each case Fragment **1** was found to adopt a similar orientation (Fig 5A). The large variation in the position of the amino-triazole ring position relative to the average structure of Fragment **1** in the model is most likely due to the lack of intermolecular NOE distance constraints to this portion of the molecule. Of the 200 conformers, we took the 10 best conformers with the lowest HADDOCK scores generated from weighted summations of energies. A representative model structure of the lowest energy conformer of the complex is shown in Fig 5B.

For all crystallization experiments we used the E82I variant of PaDsbA1. Crystal soaking experiments yielded a 1.45 Å resolution co-crystal structure of PaDsbA1-E82I in complex with Fragment **1** with an R_factor/_R_free_ of 13.6%/16.3% respectively. The quality of the electron density maps and Molprobity values indicate that the final structure is of very high quality. Details of data collection, solution methods and additional quality indicators are given in Tables 2 and 3.

**Table 2:** X-ray crystal structure of PaDsbA1 and Fragment 1: Data collection and processing.

Wavelength (Å)	0.95370
Resolution range (Å)	35.10–1.45 (1.47–1.45)
Space group	P 2_1_
Unit cell (Å, °)	35.47 63.10 41.87 90 98.31 90
R_merge_	0.077 (1.009)
Total number of observations	232,922 (10,234)
Total number unique	31,349 (14,514)
Mean((I)/sd(I))	14.1 (2.3)
Mn(I) half-set correlation CC(1/2)	0.998 (0.808)
Completeness (%)	96.4 (92.1)
Multiplicity	7.4 (7.1)

Values in parentheses refer to the highest resolution shell.

**Table 3:** X-ray crystal structure of PaDsbA1 and Fragment 1: Refinement and model quality.

**Refinement**	
R-factor (%)	13.59 (19.45)
R-free^§^ (%)	16.32 (24.44)
Number of atoms	3275
macromolecules	1,518
ligands	51
water	141
Protein residues	187
**R.M.S.D from ideal geometry**	
RMS(bonds) (Å)	0.008
RMS(angles) (°)	1.15
**Molprobity analysis**	
Ramachandran favored (%)	98.5
Ramachandran outliers (%)	0
Clashscore*	3.2 (97^th^ percentile; N = 479, 1.447Å ± 0.25Å)
Molprobity score**	1.1 (98^th^ percentile; N = 3441, 1.447Å ± 0.25Å)
Average B-factor (Å^2^)	19.20
macromolecules	17.50
solvent	32.50

Values in parentheses refer to the highest resolution shell

^§^ R_free_ calculated over 5.0% of total reflections excluded from refinement

* Clashscore: 100^th^ percentile is the best among structures of comparable resolution; 0^th^ percentile is the worst. For clashscore the comparative set of structures was selected in 2004, for MolProbity score in 2006

** MolProbity score combines the clashscore, rotamer, and Ramachandran evaluations into a single score, normalized to be on the same scale as X-ray resolution

### Fragment 1 binds to the non-catalytic face of PaDsbA1 at the domain interface

**Fig 6:**
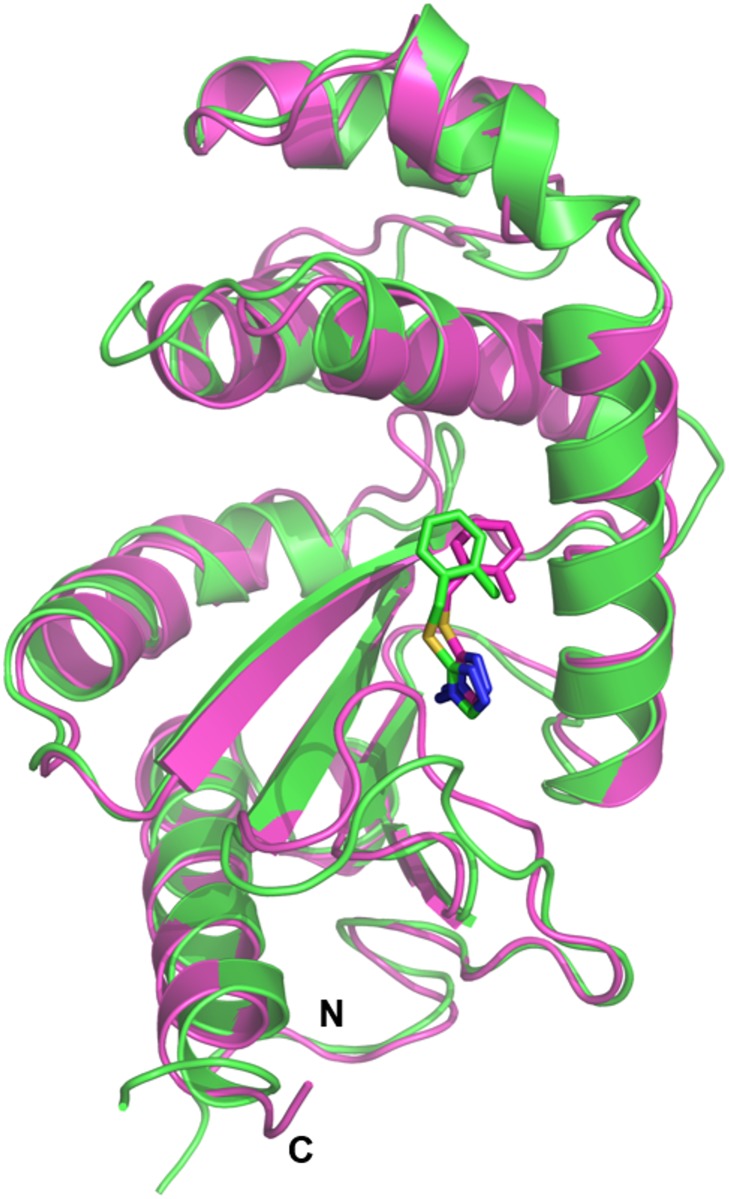
NMR data driven HADDOCK docking model and X-ray crystal structure of PaDsbA1–Fragment 1 complex. The representative HADDOCK model and the crystal structure are shown in cartoon representation coloured green and magenta, respectively. The binding mode of Fragment **1** (shown as sticks) is similar in both models.

**Fig 7:**
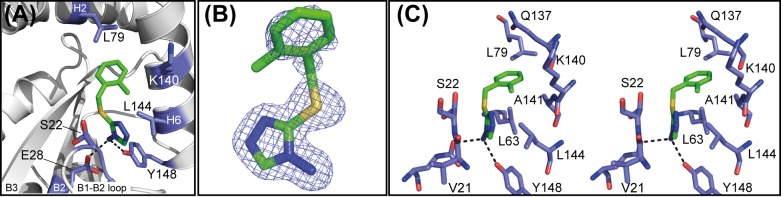
X-ray crystal structure of PaDsbA1-Fragment 1 complex. The structure of PaDsbA1 in complex with Fragment **1** was solved by X-ray crystallography. **(A)** Residues from both the helical (H2, H6) and TRX (B2, B1-B2, H6) domains contribute to the binding of Fragment **1**. Selected side chains, which make contact with Fragment **1** are shown as sticks, and hydrogen bonds identified in the complex as dashed black lines. **(B)** 2Fo-Fc (blue) electron density map for Fragment **1**, was generated from a simulated annealing omit map and is shown contoured at 1.0 σ. The maps are shown within a 2 Å radius of each atom of Fragment **1**. **(C)** Stereo representation highlighting the subset of side chain residues involved in either hydrogen bond or hydrophobic contacts with Fragment **1** in the complex. Hydrogen bonds are shown as black dashed lines.

In both the X-ray structure and the HADDOCK models of the complex, Fragment **1** is positioned at an interface between the thioredoxin (TRX) and helical domains of PaDsbA1, on the non-catalytic face of the enzyme (Fig 6). The groove in which Fragment **1** binds to PaDsbA1 is extensive and straddles the boundary between the TRX and helical domains. An analogous surface in the structure of EcDsbA was described as “Groove 2” [42]. Fragment **1** bound to PaDsbA1 at the end of this groove adjacent to helix H6 (Fig 7A). The amino-triazole ring of Fragment **1** binds into a pocket that is formed by residues Tyr148, Ser22 and Glu28, where it is sandwiched between the surface formed by helix H6, the B2 sheet and B1-B2 connecting loop (Fig 7A) The *ortho*-methylbenzene group is positioned close to the interface of H6 and H2 of the helical domain. Residues from both the helical and TRX domains contribute to the binding of Fragment **1**; H6 straddles the TRX and helical domains of PaDsbA1 and residues from each domain make contacts with the fragment.

The NMR and crystal structures provide complementary information about the molecular interactions that dictate Fragment **1** binding to PaDsbA1. In the NMR structure, there are no NOE-based constraints for the 5-membered triazole ring, which has only one non-exchangeable proton. In the crystal structure however, the majority of observable contacts between PaDsbA1 and Fragment 1 are to this amino-triazole group for which the electron density map is sharply resolved (Fig 7B). Specifically Fragment **1** is coordinated by two side-chain mediated hydrogen bonds between the amino substituent of the triazole ring and residues Glu28 (2.99 Å) and Tyr148 (2.92 Å). Additional stabilization is provided by a π-stacking interaction between the triazole ring and amide bond of Val21 –Ser22 (3.55 Å from centroid to Ser22 Cα).

Electron density for the *ortho*-methylbenzene group is less well defined in the X-ray structure (Fig 7B) presumably the result of a degree of flexibility. It is positioned within the vicinity of Leu63, Leu79, Gln137, Ala141, Lys140 and Leu144 (Fig 7A and 7C), which is well supported by NMR data (Fig 5B). The crystal structure of PaDsbA1 undergoes very minor conformational changes upon fragment binding. Notably the position of Tyr148 –which makes the key hydrogen bonding interaction with Fragment **1** –is unaltered between the *apo* and ligand bound structures. There are a number of minor changes however; the side chain of Gln137 flips in the liganded structure, forming a new contact to a water molecule. Ser22 OH adopts two alternate side chain orientations in the Fragment **1** bound structure. Finally, the main chain atoms of Val61 deviate between the two structures (regardless of how a superposition is performed), although the reasons for this are unclear.

## Discussion

Our fragment screen of the oxidoreductase enzyme PaDsbA1 identified 8 fragments with measurable K_D_ values of < 5 mM. The ligand efficiencies (LEs) of these fragments range from 0.19–0.37 kcal mol^-1^ HAC^-1^ and lipophilic ligand efficiencies (LLEs) range from 0.06–0.44 kcal mol^-1^ HAC^-1^ [17]. Together, the hit rate observed in the screen, the binding efficiency of the ligands identified and the availability of a structure of Fragment **1** in complex with PaDsbA1 suggest that this pocket may be amenable to the development of more potent binders.

The three most potent hits produce CSP patterns in HSQC spectra that are consistent with an unexpected mode of binding at the non-catalytic face of PaDsbA1. This was verified for Fragment **1** in both solution-NMR and X-ray crystal structures. We recently described a classification of DsbA proteins that broadly divides the enzyme family into two classes on the basis of β-sheet topology in the TRX domain [43]. These classes (DsbA-I and DsbA-II) can be further subdivided by surface features, specifically the conformation and composition of three loop regions on the active site surface of the enzyme and electrostatic features [43]. PaDsbA1 is a DsbA-I protein. DsbA-I proteins have a groove that runs along their non-catalytic face between the TRX and inserted helical domain. In this study, fragments bind within this groove on the non-catalytic face. To our knowledge this is a previously unreported site of specific small molecule binding to DsbA proteins, although small molecules such as glycerol and DMSO (present in the crystallization condition or introduced via soaking) have been resolved in the equivalent groove of other DsbA-I structures [3, 44]. The non-catalytic face of DsbA-I enzymes has also been identified previously as a protein interaction surface. In *Acinetobacter baumannii* DsbA (AbDsbA) the groove can accommodate an elongated peptide chain as determined by a crystal structure of AbDsbA in complex with *E*. *coli* Elongation Factor EF-Tu [45]. Whilst the physiological relevance of this particular protein-protein interaction remains to be determined, it suggests that the groove may serve as an additional protein interaction site in at least DsbA-I type enzymes.

Overall, our success rate in obtaining structural information about fragment-protein interactions was low. Of 45 validated hits from a screen of 1137 fragments, we obtained a crystal structure for a single fragment-protein complex. This result reflects the inherent difficulties of forming liganded crystal structures at the start of a fragment campaign when hit compounds are frequently of low affinity (Fig 2). The challenge is compounded further when targeting the relatively flat and featureless surfaces that often typify protein:protein interactions [46] such as that of PaDsbA1. Even against more classically druggable targets with active site pockets and cavities, translating well-validated hits into high-resolution structural information is not always straightforward. Targeting three enzymes, Hubbard *et al* reported that co-crystal structures were obtained for 10, 17 and 81% respectively of “class I” NMR fragment hits against each target, where a class I hit was defined as a fragment that bound competitively to the target in each of three separate ligand-detected NMR experiments [47]. Similarly, targeting *Mycobacterium tuberculosis* pantothenate synthetase, Silvestre *et al*., utilized a multi-step biophysical investigation and validation of hits (thermal shift, Water LOGSY NMR, STD NMR and ITC) and yet obtained crystal structures for less than half (47%) of the validated hits, Notably, all of these hits were those for which ITC gave K_D_ values of 5 mM or less, supporting the idea that affinity and the resultant binding site occupancy at early stages of ligand-structure determination is important [48].

The inability to obtain fragment-target structural information—particularly high-resolution crystal structure information—is a significant bottleneck in fragment-based drug design (FBDD) campaigns. Yet structural information is very important. A review of 145 FBDD programs found that 62% used structural information (10% NMR, 52% X-ray) to select the best fragments for elaboration and 77% of fragment-derived clinical candidates involved structure based optimizations [49]. Some campaigns have been able to elaborate fragments entirely in the absence of structural information. Inhibitors of FtsZ (a protein involved in bacterial cell division) were progressed from initial hits to highly potent drug like molecules solely using *in vitro* antibacterial assays and bacterial cell morphology analysis to guide medicinal chemistry [50]. However, this is an unusual case in that the starting ~110 Da fragment already displayed *in vitro* antibacterial activity. A more generic approach allowing elaboration without an explicit requirement for structural characterization is the “off-rate screening” approach using surface plasmon resonance reported by the FBDD group at Vernalis [51]. NMR guided models provide an alternative, if lower-throughput, option for structural information, although a number of approaches to improve the speed of structure determination of similar complexes have been reported [30, 52, 53]. In the case of PaDsbA1—Fragment **1**, an accurate model of the complex was derived from sparse NOE data. Protein—fragment structure solution by NMR is thus a valuable adjunct to X-ray crystallography in the FBDD process, and in cases where crystallography is intractable can serve as a practical alternative to X-ray methods.

It remains to be seen whether targeting the non-catalytic face of PaDsbA1 is a viable means of inhibiting PaDsbA1 function. PaDsbA1 undergoes very minor structural changes upon Fragment **1** binding. Interestingly, binding of AbDsbA to *E*.*coli* EF-Tu (which engages AbDsbA along an equivalent groove on the non-catalytic face), significantly reduced AbDsbA enzymatic activity in an *in vitro* model-peptide folding assay [45] suggesting a potential for inhibition by targeting this face of this protein. However, Fragment **1** was found to have no inhibitory activity in the same *in vitro* model-peptide folding assay even at final concentrations of 10 mM. Development of allosteric inhibitors from fragment campaigns is not without precedent [54]. Whether a more potent analogue may have inhibitory effects via allosteric inhibition remains to be investigated.

Finally, we note that two DsbA enzymes, PaDsbA1 and PaDsbA2 have been identified in *P*. *aeruginosa*. The second enzyme PaDsbA2 is a highly oxidizing DsbA of undetermined function [55]. PaDsbA1 is the primary oxidase *in vivo*, and cannot be complemented by PaDsbA2 suggesting that the two proteins have different substrate specificities. Furthermore, PaDsbA2 appears to be expressed only under specific, as yet unidentified, conditions suggesting a highly specific role for PaDsbA2 in *P*. *aeruginosa* physiology. In light of the role of PaDsbA1 as the primary disulfide donor in *P*. *aeruginosa*, and as all *in vivo* and animal model work to date has focused on PaDsbA1, we have focused on the identification of PaDsbA1 inhibitors which are anticipated to disrupt normal *P*. *aeruginosa* oxidative folding activity.
